# COVID-19 pandemic and gynaecological laparoscopic surgery: knowns and unknowns

**Published:** 2020-04-01

**Authors:** R Mallick, F Odejinmi, TJ Clark

**Affiliations:** Princess Royal Hospital, Brighton and Sussex University Hospitals NHS Trust, Lewes Road, Haywards Heath, RH16 4EX, UK;; Whipps Cross Hospital, Barts Health NHS Trust, Whipps Cross Road, Leytonstone, London, E11 1NR, UK;; Department of Obstetrics and Gynaecology, Birmingham Women’s and Children’s Hospital,Birmingham, B15 2TG, UK.

**Keywords:** COVID-19, coronavirus, surgery, laparoscopy

## Abstract

The worldwide impact of COVID-19 continues to be felt as hospitals in all countries reduce elective and non- urgent cases to allow staffing and resources to be deployed elsewhere. Urgent gynaecological and cancer procedures are continuing, and it is imperative all theatre staff are protected and risks of SARS-CoV-2 viral transmission reduced when operating on asymptomatic, suspected or confirmed COVID-19 patients. In particular, there are concerns relating to the transmission of COVID-19 during gynaecological laparoscopic surgery, arising from the potential generation of SARS-CoV-2 contaminated aerosols from CO_2_ leakage and the creation of smoke from the use of energy devices. The aim of this paper is to review all the up to date evidence, including experiences from China and Italy, to guide the safe management of such patients when undergoing gynaecological procedures.

## Background

### 

Since the swift arrival of COVID-19, most national bodies and learned societies have advised the urgent suspension of elective surgery with the focus shifting to emergency and cancer surgery (ACOG, 2020). This allows staffing and key resources to be deployed to where they are most required (RCOG, 2020). However, in those continuing to undertake emergency gynaecological and oncological surgical procedures, it is imperative that precautions are taken in patients who are potential, or proven, COVID-19 cases to reduce the risks of inadvertent viral transmission.

### Viral transmission in surgically generated smoke and aerosols

SARS-CoV-2 virus is largely transmitted via respiratory droplets and the highest transmission risks arise when undertaking aerosol generating procedures (AGPs) i.e. medical and patient care activities that can result in the release of airborne particles (aerosols). AGPs can create a risk of airborne transmission of infections that are usually only spread by droplet transmission. Due to the high viral load in respiratory secretions, intubation and extubation remain one of the highest risk AGPs (Meng et al., 2020). The main at-risk healthcare professionals are thus anaesthetists and emergency department clinicians and guidance is available in the wider literature to safeguard these clinicians and reduce transmission risks (Wong et al., 2020). Many emergency laparoscopic procedures will still be required during this pandemic, however very little is known regarding the risks to the health care professions undertaking these procedures. The aim of this paper is to review all the up to date evidence, including experiences from China and Italy and extrapolated data from previous outbreaks, to guide the safe management of suspected and positive COVID-19 patients when undergoing gynaecological procedures.

In laparoscopic surgery, the creation of an artificial pneumoperitoneum may increase the risk of aerosol exposure to the whole operating team. Specific evidence relating to the presence of SARS-CoV-2 virus in the peritoneal cavity and thus its transmission during laparoscopic surgery is lacking, but theoretical risks may be extrapolated from previous pandemics and viral infections. For example, prior to COVID-19, previous studies have shown that other pathogens such as activated corynebacterium, human papillomavirus (HPV), hepatitis B virus (HBV) and human immunodeficiency virus (HIV) have been detected in surgical smoke (Capizzi et al., 1998, Hensman et al., 1998, Johnson and Robinson 1991, Alp et al., 2006).

The presence of HPV DNA has been reported in approximately 40% of smoke plumes following loop excision biopsy of the cervix (Sood et al., 1994). An analysis of surgical smoke in HBV positive patients undergoing operative laparoscopic procedures, found the presence of HBV in over 90% of cases (Kwak et al., 2016). The risk of disease spread therefore theoretically exists with surgical smoke, however actual documented cases are rare with only 4 confirmed cases of HPV transmission documented in the wider literature (Liu et al., 2019). No cases of HIV or HBV transmission have been documented (Bree et al., 2017). However the presence of these pathogens are not an absolute contraindications to laparoscopic surgery as long as universal infection control precautions are undertaken (Diettrich and Kaplan 1991, Eubanks et al., 1993).

### Energy modalities

Both ultrasonic and electrosurgical devices have the propensity to create large surgical plumes thus potentially increasing the risks of viral transmission. Recent NHS pandemic Coronavirus Infection Prevention and Control guidance states that high speed surgical devices generate aerosol (DHSC, 2020). Ultrasonic devices are high frequency oscillating devices which may hypothetically add to the potential risk although the magnitude of any such risks are unknown (Zheng et al., 2020).

### Operating theatre environment

Most standard operating theatres have a positive pressure relative to the surrounding air (e.g. in corridors and adjacent areas) to prevent the flow of air from less sterile areas into a more sterile one. However, this positive pressure environment created within the operating theatre makes the spread of aerosols faster, posing an increased airborne viral transmission risk.

A negative pressure environment is ideal to reduce dissemination of the virus beyond the operating theatre although such facilities are not widely available. A high frequency of filtered air exchanges may help reduce viral load within an operating theatre (Wong et al., 2020).

### Open vs. laparoscopic surgery

It should be borne in mind that the risks of airborne viral transmission are not restricted to laparoscopic surgery because both open and laparoscopic surgery have the propensity to generate aerosols. However, the overall risk may be lower with open surgery (Li et al., 2020) as an artificial pneumoperitoneum is not created and maintained. During laparoscopic procedures, the risk of aerosol generation is increased during insertion, and particularly removal, of trocars from a pressurised peritoneal cavity. This can result in dispersion of body fluids especially where port sites are contaminated with blood or other human tissues.

As this pandemic progresses, the need to continue operating in emergency situations such as ectopic pregnancies on suspected or confirmed COVID-19 patients will increase.

Hypothetical risks of viral transmission must be balanced against the significant benefits of adopting a minimally invasive approach, namely reduced morbidity and mortality, shorter hospital stay and quicker return to daily activities (Snyman et al., 2017, Aarts et al., 2015).

These advantages have gained even more significance in helping to maintain wider public health, in light of the pressure on health care resources imposed by this pandemic.

The evidence so far does not suggest an increased risk of COVID-19 transmission during gynaecological laparoscopic surgery when personal protective equipment (PPE) is used.

Molecular studies have detected viral RNA in a range of bodily specimens from COVID-19 patients, including upper and lower respiratory tract samples, faeces and blood, indicating the potential presence of infectious virus. SARS-CoV-2 virus has been detected in faeces in 29% of cases (Wang et al., 2020), presumably thorough transmission from the naso-pharynx with ingestion into the gastrointestinal tract. However, it is less commonly found in blood, with studies reporting viral RNA positive samples in 1-15% of COVID-19 patients (Wang et al., 2020, Chen W et al., 2020, Young et al., 2020, Chan et al., 2020, Huang et al., 2020). The virus has not been found in the female genital tract in women with proven COVID-19, although the data are few (Chen Y et al., 2020, Fan et al., 2020). Hence operations carried out by other specialities such as ENT and upper gastrointestinal endoscopy are at a higher risk of generating aerosols and have more significant safety concerns (RCS, 2020). Moreover, bowel surgery may have different implications compared to gynaecological interventions. Thus, there is currently insufficient evidence to support adopting a universal open approach to surgery for gynaecological indications.

The Royal College of Obstetricians and Gynaecologists (RCOG) along with the British Society for Gynaecological Endoscopists (BSGE) recently released a statement on gynaecological laparoscopic procedures and COVID-19. They recommended that laparoscopic approaches should be utilised when feasible in preference to laparotomy (BSGE, 2020). These sentiments have been largely echoed by both the European Society for Gynaecological Endoscopy (ESGE, 2020) and American Association of Gynecologic Laparoscopists (AAGL, 2020). Furthermore there still remains a paucity of data surrounding the safety of the open approach and the potential transmission risks, including the use of electrosurgery. However this situation is ever evolving and the advice may change as the consequences of COVID-19 infection and its methods of transmission becomes more greatly understood.

### Personal protective equipment

It is imperative all medical and theatre personnel are protected during this pandemic and full precautions are taken to reduce the transmission risks. Inadequate protection results in an increased infection rate amongst medical and nursing staff with significant morbidity and in some cases sadly mortality, placing a further strain on already struggling health services when resources are at a premium. It is important to follow both local and national (DHSC, 2020) guidance where available as regards the recommended level of PPE and keep abreast of any modifications.

## Recommendations for laparoscopic surgery in suspected or confirmed COVID-19

**Figure 1:**
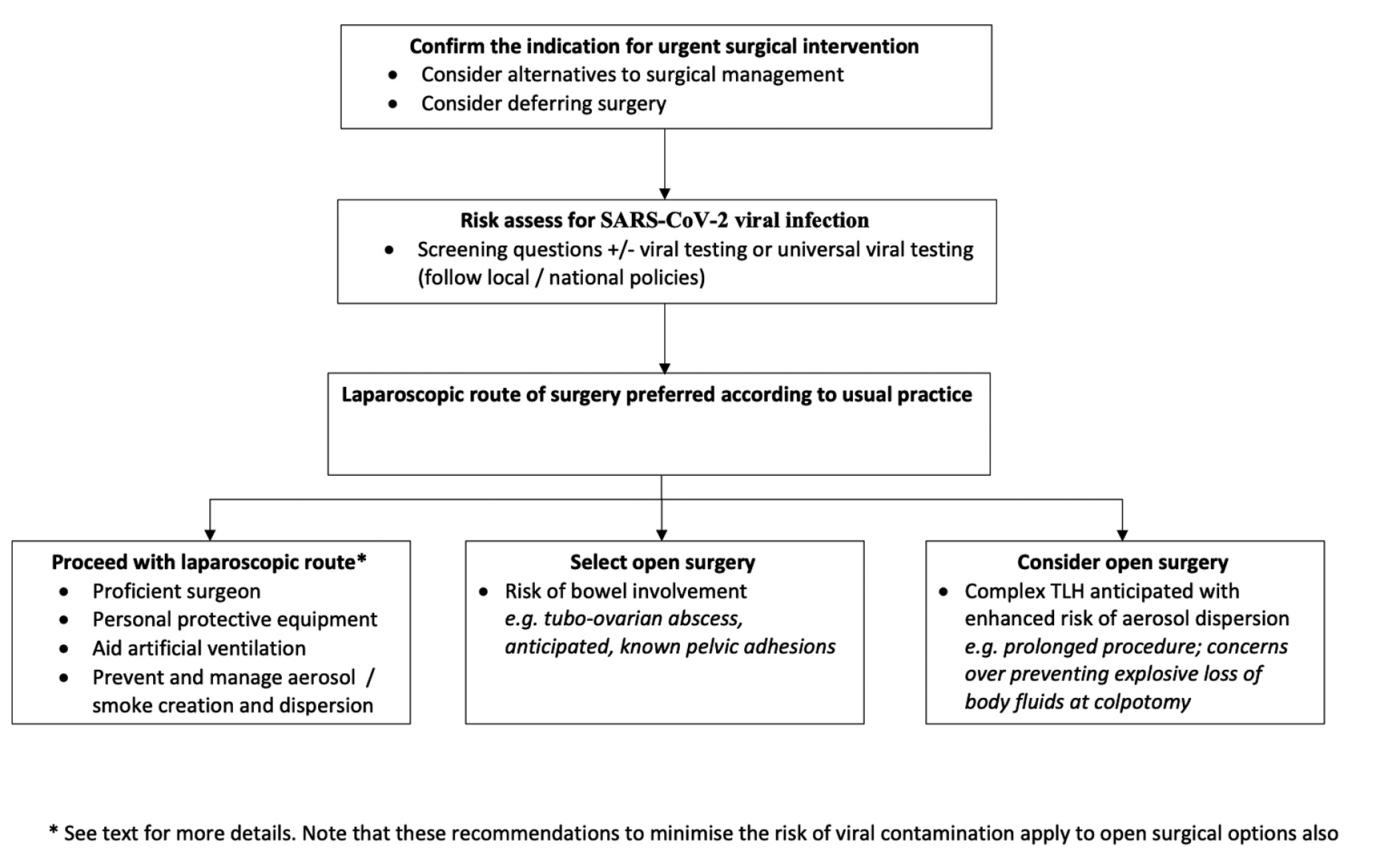
Recommendations on Gynaecological Laparoscopic Surgery during Covid 19

There remains a paucity of evidence surrounding SARS-CoV-2 virus transmission particularly in laparoscopic gynaecological surgery, however these recommendation are based on national and international guidance and extrapolated data from other viral infections (BSGE, 2020, ESGE, 2020, AAGL, 2020). These principles should be followed, in suspected or confirmed COVID-19 patients, to reduce the potential transmission risk (Figure 1). The safety of all members of the team is of the utmost importance and can be facilitated by operating in a safe, structured environment.

1. Non-surgical treatments should be utilised where possible to reduce the risk of horizontal transmission of SARS-CoV-2 virus to health care workers, and reduce the need for hospital admission, provided they are a safe alternative.
2. Patients should be risk assessed for potential SARS-CoV-2 viral infection. Consider universal SARS-CoV-2 virology screening in all patients undergoing surgery. Patients testing negative can proceed with the standard laparoscopic technique and routine surgical infection control procedures.
3. If urgent surgery is required and testing is not possible, manage as suspected COVID-19 with precautions described.
4. Hospitals should have clear pathways and make theatre allocations to separate
  - COVID-19 positive patients
  - Clinical emergencies
  - Clinically urgent cases
  - Trauma patients
5. The laparoscopic procedures should be undertaken by the most experienced surgeon available to ensure full knowledge of safe laparoscopic procedures are followed and to ensure that the procedure is performed in the shortest time possible.
6. To protect operating staff:
  - Enhanced PPE is mandatory for all theatre personnel (i.e. disposable gloves, disposable fluid- resistant gown, filtering face piece class 2 or 3 or N95 respirator and disposable eye protection)
  - In order to minimise possible exposure and to guard against depletion of PPE stock, ensure that only staff that are required for the procedure are present in theatre
  - Theatre staff need to be versed in self-protection strategies against suction materials and blood contaminated areas in theatres and in laparoscopic suction in line with local protocols
7. To aid artificial ventilation
  - Operating pressures should be kept as low as possible
  - Minimise the amount of Trendelenburg
8. To prevent and manage aerosol dispersion
  - Caution and care should be taken during insufflation
  - Special attention should be paid to port sites to prevent explosive dispersion of body fluids both at the insertion/removal of trocars and specimen retrieval
  - Limit the number of incisions where possible, although there should be enough port sites to allow safe and expeditious surgery
  - Ensure that incisions are of appropriate size to prevent leakage during the procedure
  - Minimise exchange of instruments to minimise leakage
  - Caution when using ultrasonic devices as the potential for aerosol generationmay be higher.
  - Employ electrosurgical and ultrasonic devices in a manner that minimises surgical smoke production with low power settings and avoidance of prolonged activation
  - Suction devices, smoke evacuation filters, retrieval devices and swabs should be used to: (1) prevent aerosol transmission: remove smoke, aerosol and the CO_2_ pneumoperitoneum during surgery and (2) avoid explosive dispersion of body fluids when removing trocars and retrieving specimens.
9. Only evacuate surgical smoke via the tap on ports when attached to a smoke evacuation filter and / or by direct suction using a vacuum suction unit.
10. Only evacuate the pneumoperitoneum via direct suction using a vacuum suction unit.
11. Gynaecological operations that carry a risk of bowel involvement, however small should be performed by laparotomy.

## Specific surgical considerations

### Deflation technique at the end of a procedure

Standard laparoscopic practice is to remove all ports under vision and subsequently expel all the operative gas followed by removal of the laparoscope under direct vision. In view of the potential aerosol risks all ports used to release gas should have a suitable smoke extraction device attached. At the end of the procedure, gas should be removed using the combination of a suction device and releasing gas slowing via a filtered port. The accessory ports should be removed slowly and over a blunt probe, which is subsequently removed, to reduce the risk of hernia as removal under direct vision is not possible when avoiding inadvertent gas leakage.

The primary port should still be removed under direct vision once the abdomen has been completely deflated.

Ports larger than 5mm should be closed as per standard practice, however a J needle closure (after all other ports are removed) is recommended rather than using an Endoclose device, which would increase the risk of gas escaping from the abdomen.

### Total laparoscopic hysterectomy (TLH)

Laparoscopic hysterectomies, in the context of malignancies, are likely to continue to be undertaken in this pandemic. Particular care must be taken during colpotomy and subsequent uterine extraction to minimise unfiltered gas leakage and sudden loss of blood and other body fluids. The abdomen should be emptied of gas using a suction device and filtered ports, as described above, prior to removal of the uterus. To minimise airborne droplet transmission consideration should be given to performing an open hysterectomy, on a case by case basis.
